# Characterization of kitchen and municipal organic waste for biogas production: Effect of parameters

**DOI:** 10.1016/j.heliyon.2023.e16360

**Published:** 2023-05-16

**Authors:** Register Mrosso, Achisa C. Mecha, Joseph Kiplagat

**Affiliations:** a Renewable Energy, Nanomaterials, and Water Research Group, Department of Mechanical, Production &Energy Engineering, Moi University, P.O.Box 3900, Eldoret, Kenya; b Renewable Energy, Nanomaterials, and Water Research Group, Department of Chemical and Process Engineering, Moi University, P.O. Box 3900, Eldoret, Kenya; c Department of Mechanical, Production & Energy Engineering, Moi University, P.O. Box 3900, Eldoret, Kenya; d Clean Energy Technologies Research Group, Department of Materials, Energy Science and Engineering, Nelson Mandela African Institution of Science and Technology (NM-AIST), P.O. Box 447, Arusha, Tanzania

**Keywords:** Characterization, Total solids, Volatile solids, Biogas yield, Cooked rice, Cabbage

## Abstract

Globally, the production of municipal solid waste is rising annually because of consumerism and the urbanization process. In the past few years, different researchers have explored strategies for generating biogas from various organic wastes. In this study, kitchen waste and municipal solid waste were characterized by several physical-chemical parameters. Ten of these substrates were mono-digested for biogas production in batch reactors where cabbage showed a 96.36 ± 1.73% volatile solid and biogas yield of 800 ± 8.8 mL within 10 days, while cooked rice had an 83.00 ± 1.49% volatile solid, and a biogas yield of 2821 ± 31.03 mL within 28 days. The CN ratio for cabbage and cooked rice waste was 13.9 and 30.9 respectively, whereas their pH values were 6.2 and 7.2. Based on the characterization and biogas yields attained, cooked rice waste could be mono-digested for biogas production and no published work showed a high yield as the current study while the other substrates require co-digestion to improve the biogas yield.

## 1 Introduction

The threat of climate change due to the emission of greenhouse gases from conventional energy resources, foreseen exhaust of non-conventional energy sources, and the fluctuation of petroleum products cost have spurred the exploration of renewable energy resources [1]. Using non-conventional energy is one of the strategies most countries adopt to mitigate the problems associated with using non-renewable energy sources. The generation of kitchen waste and municipal solid waste increases the operation cost of households and municipal councils due to transportation and disposal costs [2].

Worldwide, the production of municipal solid waste is increasing yearly accompanied by consumption standards and urbanization. The emergent countries release 109.5–525.6 kg of municipal solid waste (MSW) person per year whereas industrialized nations typically produce 521.95–759.2 kg of municipal solid waste per person per year [3]. Globally, MSW generation exceeds 2 billion tons per annum that threaten the environment [3]. Since these wastes have been found to harm the environment, there are urgent measures needed to eliminate them from the surrounding. Thus, various ways have been devised for their removal, and they include decomposing, incineration, and landfill [4]. However, all these methods have been found to negatively affect the environment as they pollute the environment and release greenhouse gasses, thus leading to climatic changes, which are now an agenda worldwide.

Anaerobic digestion (AD) is widely utilized to address organic waste management in households, industries, and urban areas [5] thus; overcoming the challenges of energy insecurity and waste management. The application of AD reduces greenhouse gases emission by utilizing municipal solid waste and kitchen waste for biogas yield, nutrient recycling, and energy recovery from digested biomass [6]. During anaerobic digestion, bacteria degrade the organic matter found in kitchen waste (KW), and municipal solid waste (MSW) to generate the energy required for their consumption, survival, and biogas. Many solid wastes such as Irish potato peels, cabbage waste, and cooked rice waste are produced and their effectual getting rid of is a matter of concern. Meanwhile, these organic wastes from KW and MSW have great potential for biogas production. Bioconversion processes are suitable for the substrate that contains a moisture content of more than 50%. A major problem of AD in fruit and vegetable waste is the speedy acidification due to low pH and a higher level of producing volatile fatty acid, which therefore limits methanogenation in the digester. The efficiency of the process and the amount of biogas generated depends on total volatile solids (TVS), moisture content (MC), total solids (TS), type of substrate used, hydraulic retention time (HRT), pH, carbon-nitrogen (CN) ratio of the substrate, and the presence of toxic metals in the substrate [7].

Moisture content enhances the growth of methanogenic bacteria and facilitates their movement and transportation of nutrients. Total volatile solids and total solids give useful details regarding the amount of biogas, which will be produced, and the efficiency of the anaerobic process. High volatile solid content may not necessarily be transformed into high biogas yield as there is the existence of inadequate volatile solids of lignin material [8]. For instance, a study by Sajeena et al. [9] reported maximum biogas yield when TS is about 10% and moisture content of about 90% while Tsunatu et al. [8] reported a high yield of 9% total solids and moisture content of 91%. Anaerobic digestion plays an important role in environmental and agricultural sustainability as an effective method for waste stabilization in converting bio-waste into sustainable energy and nutrients rich digestate. Biogas is produced in bio-waste digesters, sewage sludge, and landfills during the anaerobic breakdown of organic matter [10]. Biogas contains methane (55–77%), carbon dioxide (30–45%), hydrogen sulfide (1–2%), nitrogen (0–1%), hydrogen (0–1%), siloxanes, water vapor, a trace of oxygen, and carbon monoxide [11].

Not all wastes are suitable for biogas production, and sometimes yield might not be viable. Therefore, in evaluating the feasibility of the substrate for biogas production, characterization is necessary. The present work, characterized kitchen waste and municipal solid waste substrates (moisture content, volatile solids, total solids, electrical conductivity, total dissolved solids, nitrates, and biodegradability index) as well as mono-digestion to establish the outcome of the feedstock characteristics on biogas production. The significance of this study entails in the utilization of municipal solid waste and kitchen waste for biogas yield. The study addresses the setbacks of energy insecurity and waste disposal as waste management and becomes a key topic in environmental protection and management.

## 2 Material and methods

### 2.1 Materials and equipment

Different kitchen wastes (KW) were obtained from the cafeteria in Moi University campus; cooked rice (CR), cooked banana (CBN), cooked beans (CB), cooked Irish potatoes (CIP), and ugali (UG), were used. The MSW were collected from the market around Moi University which include banana peels (BP), kale (SWW), spinach leftover (SW), cabbage leftover (CBG), pineapple peels (PNP), tomato (TMT), carrot peels (CP), and African nightshade (AFNT), and Irish potato peel (IPP). The following equipment was used; crucibles, electrical blender, electronic precision balance (model HZT-A200), laboratory oven (model LDO-150), and muffle furnace (ELF11/14B 220–240V 1 PH + N). Similarly, the Hatch multi-parameter (model HQ40d), spectrophotometer (DR-900), and a BOD incubator (model WTW™ 208432) were used for physical-chemical analysis.

### 2.2 Methods

Moisture content, volatile solids and total solids were calculated gravimetrically in an oven-drying ignition method following standard formula reported in Ref. [12].

#### 2.2.1 Calculation of feedstock's moisture content

Moisture content is the mass of water held in the material; it is normally indicated as a percentage of weight. The crucible was accurately washed and drained in an oven at 105 °C for about 30 min and left in an oven for cooling to ambient temperature. The empty dried crucible was weighed before use (W_1_). A wet sample of each substrate was added to the crucible and carefully placed in an oven under 105 °C for about 3 h to a constant weight (W_2_). The crucible and the feedstock were permitted to cool in the oven to ambient temperature, weighed again using a mass balance (W_3_). The determination of the moisture content was done using Equation (1) as described in Ref. [13].

(1) $$MC=\frac{\left(W_{2}-W_{1}\right)-\left(W_{3}-W_{1}\right)}{\left(W_{2}-W_{1}\right)}\times 100\%$$

#### 2.2.2 Determination of total solid (TS)

The total solid is the quantity of solid available in the feedstock following the disappearance of water or the amount of organic matter remaining in the crucible following the vaporescence process. The drying process was done in an oven at 105 °C [5]. Equation 2 was applied to determine the percentage of total solids [5];

(2) $$TS=\frac{W_{3}-W_{1}}{W_{2}-W_{1}}\times 100\%$$

#### 2.2.3 Determination of total volatile solids (TVS)

**Fig. 1:**
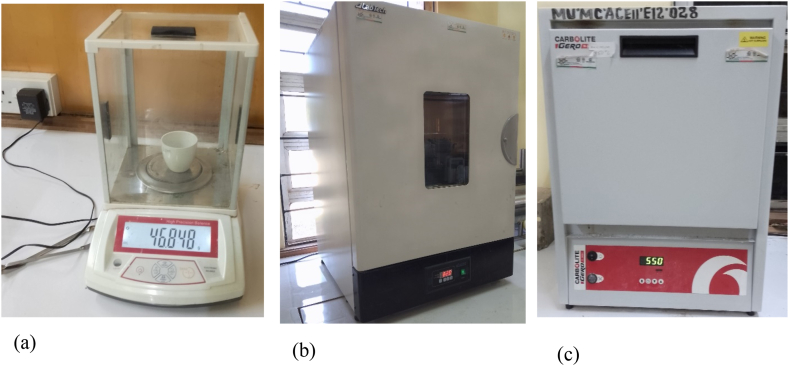
(a) Weighing balance, (b) oven, and (c) muffle furnace used in the characterization of KW and MSW samples.

The remains obtained during the calculation of TS were burnt in a muffle furnace at 550 °C for 1 h until grayish-white ash was obtained. The ignited sample and the crucible were allowed to cool in the kiln for 6 h. The sample was weighed and recorded (W_4_), and the TVS was calculated as per equation (3).

(3) $$TVS=\frac{W_{3}-W_{4}}{W_{3-}\;W_{1}}\times 100\%$$

where, W_4_ = Weight of residue after ignition + weight of crucible while %TVS = percentage Volatile total solid. The analyses were carried out in Manufacturing, Industrial, and Textile Laboratory (MIT) at Moi University campus using the equipment displayed in Fig. 1(a–c).

#### 2.2.4 Physical-chemical analysis

The analytical determination of total dissolved solids (TDS) and electrical conductivity was done using a multipara meter; nitrate (NO_3_) was quantified by Cadmium Reduction Method using Spectrophotometer DR-900. Biological oxygen demand (BOD) and chemical oxygen demand (COD) were obtained by the calorimetric procedure described by Ref. [12]. A gas analyzer (Geotech-5000) analyzed the composition of biogas. The biodegradability index (BI) was determined using equation 4:

(4) $$BI=\frac{COD}{BOD}$$

### 2.3 Experimental setup for biogas production

**Fig. 2:**
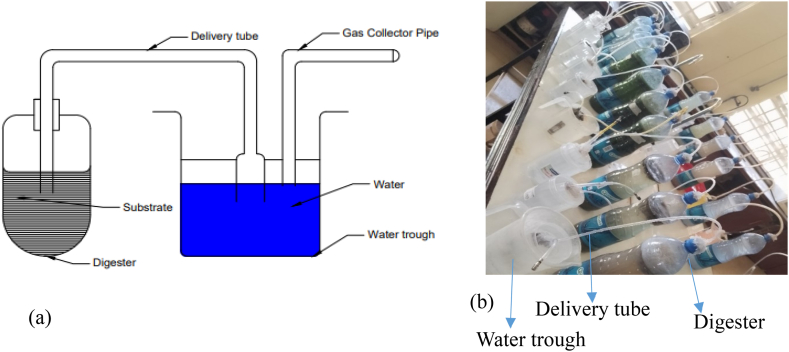
(a) A simplified diagram (b) A setup for mono-digestion for 10 substrates in duplicate.

The feedstock/substrate was blended to a constant size using an electrical blender and to enlarge the surface area to volume ratio for microbial activity. Anaerobic digestion was conducted in plastic bottle digester under batch conditions in duplicate Fig. 2(a and b). The active microbial inoculum used was collected from an active biogas plant around Eldoret town in Kenya. The biogas plant was working at mesophilic temperature using cow dung as feedstock with the existence of a large array of the highly active methanogenic community for the AD process. After feeding the reactor with the substrate it was closed tightly to avoid air entering, and the biogas generated was carefully measured through the water displacement technique, [2,14].

## 3 Results and discussion

### 3.1 Moisture content, volatile solid and total solids of the substrates

The moisture content, volatile solid and total solids of the feedstocks used in this study were obtained following the procedures indicated in Fig. 1 and equations (1), (2), (3) respectively and the data obtained are tabulated in Table 1.

**Table 1:** Moisture content, volatile solids and total solids, and for 14 feedstocks used in the current study.

	Physical-chemical characteristics (%)					
Feedstocks	MC	Ref	TS	Ref	TVS	Ref
PNP	82.00 ± 1.23	79.97 [14]	18.00 ± 0.32	15.89 [15]	95.50 ± 1.72	96.12 [16]
BP	78.30 ± 1.17	83.30 [17]	21.67 ± 0.39	19.00 [18]	94.00 ± 1.69	86.78 [17]
IPP	72.00 ± 1.08	78.00 [19]	28.00 ± 0.50	22.90 [20]	92.00 ± 1.69	93.40 [21]
CR	69.00 ± 1.04	69.72 [22]	31.00 ± 0.56	30.28 [22]	83.00 ± 1.49	90.11 [22]
UG	67.21 ± 1.01	67.80 [23]	32.79 ± 0.59	32.20 [23]	86.00 ± 1.55	95.30 [23]
CB	66.73 ± 1.00	–	33.27 ± 0.56	18.00 [24]	86.00 ± 1.55	92.80 [24]
CBN	76.00 ± 1.14	78.00 [25]	24.00 ± 0.43	22.00 [25]	94.87 ± 1.71	94.30 [24]
CIP	78.40 ± 1.78	79.00 [26]	21.60 ± 0.39	–	94.92 ± 1.71	–
CBG	92.00 ± 1.38	92.00 [27]	08.00 ± 0.14	08.00 [27]	96.36 ± 1.73	92.00 [28]
AFNT	90.32 ± 1.35	87.71 [29]	09.68 ± 0.17	12.29 [29]	81.58 ± 1.47	89.43 [29]
SW	91.70 ± 1.36	91.43 [30]	08.30 ± 0.15	08.57 [30]	76.59 ± 1.38	77.75 [30]
SWW	84.86 ± 1.27	83.00 [27]	15.14 ± 0.27	17.00 [27]	84.56 ± 1.52	–
TMT	93.43 ± 1.40	93.89 [31]	06.57 ± 0.12	06.11 [31]	82.00 ± 1.48	92.85 [18]
CP	85.00 ± 1.28	84.23 [32]	15.00 ± 0.27	15.77 [32]	88.62 ± 1.60	88.00 [33]

High moisture content in a feedstock favors the biochemical conversion process that proceeds without the addition of water hence reducing the cost contributed to water [17]. Moisture content within the substrate affects microbial activity, composite temperature, and the rate of decomposition as well as facilitates the transportation of nutrients. The moisture content of the substrates/feedstock in this study varies from 66.73 to 93.43% **(**Table 1**)** and was comparable with the literature. Tomato waste and cabbage showed the highest MC (93.43 and 92% respectively) which is favorable for high biogas yield. Cooked beans and ugali showed the lowest MC (66.73% and 67.21% respectively).

The total solid content of the substrates/feedstock used in the current study differs from 06 to 33.27% **(**Table 1**)**. From the table, it is evident that tomatoes had a minimum total solid of 06.57% while cooked beans had a maximum TS value of 33.27%. Deressa et al. [18] reported the TS for cooked rice waste to be 32.05% which is comparable with the values presented in this study. Therefore, all the results attained from this work for each feedstock were equivalent to values obtained in the literature.

The volatile solid for all samples ranges from 76.59 to 96.36%, which is alike to the values found in the literature. Spinach had the lowest TVS of 76.59% while cabbage had the highest TVS of 96.36% (Table 1). Glivian et al. [22] reported the TVS for rice waste as 90.11%, while Deressa et al. [18] reported 93.2% which is in accordance with the current work. Kafle et al. [28] reported the highest TVS for cabbage leftover was 92.00% which is in accordance with the present work.

### 3.2 Physical-chemical analysis

The physical-chemical analysis was performed for the three substrates having high volatile solid (cabbage), high CN ratio (cooked rice waste), and high total solid (ugali) and the values obtained are as per Table 2.

**Table 2:** Physical-chemical analysis of selected substrates.

	Parameter					
Substrate	TDS (mg/L)	NO_3_ (mg/L)	EC (mS/cm)	COD (mg/L)	BOD (mg/L)	Biodegradability index
Ugali	328.00	81.00	678.00	1618.00	760	2.13
Rice	805.00	54.00	16.28	1323.00	720	1.84
Cabbage	323.00	99.25	664.00	1200.50	820	1.46

Determination of the general composition of the substrate is necessary to calculate the quality and amount of biogas produced. The feedstock with COD: BOD (BI values) of 0.5 to ≤ 2:1 can be treated easily by biological means [34], this is in line with the present study whereby cooked rice waste and cabbage leftovers were in the range of 1.8:1 and 1.4:1 respectively while the ugali was observed to be 2.13:1. Therefore, it is easy for methanogenic bacteria to degrade rice waste rather than ugali and cabbage as the cabbage form volatile fatty acid easily.

### 3.3 Mono-digestion of kitchen waste and municipal solid wastes substrates

**Fig. 3:**
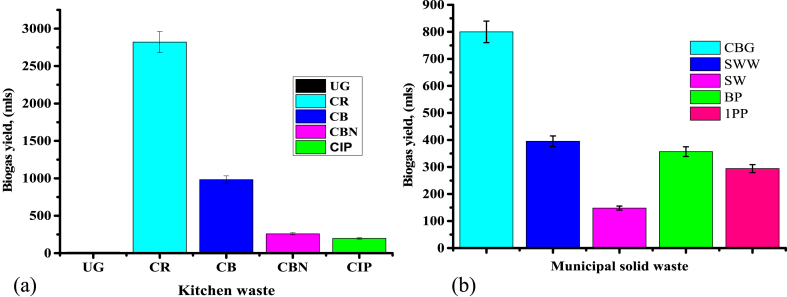
A graph of biogas yield against (a) kitchen waste and (b) municipal solid waste substrates.

**Fig. 4:**
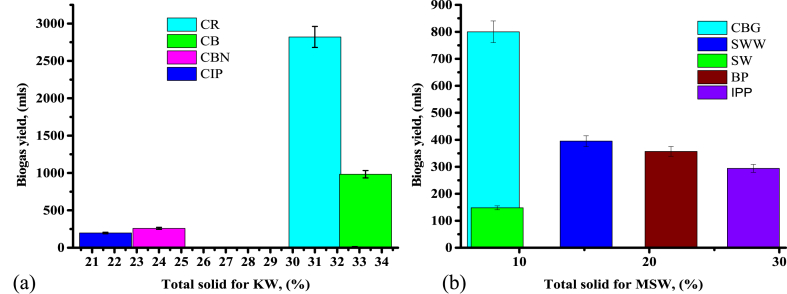
Biogas yield against TS for (a) KW and (b) MSW.

**Fig. 5:**
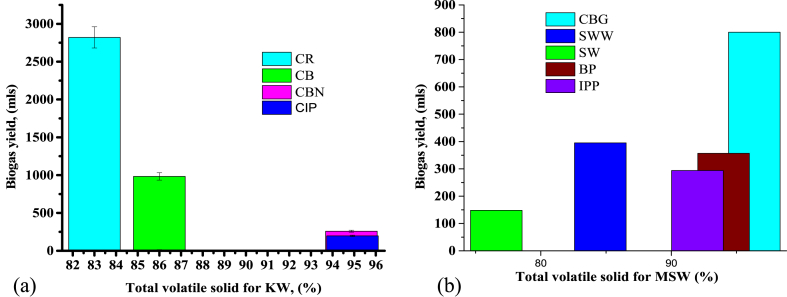
Biogas yield against TVS for (a) KW and (b) MSW.

Anaerobic fermentation of kitchen and MSW was conducted at room temperature assessing the consequences of various parameters like total solids, and volatile solids among others as discussed in the above sections, and the results are shown in Fig. 3, Fig. 4, Fig. 5.

#### 3.3.1 Biogas yield from different substrates

Fig. 3(a and b) indicates the biogas yield against feedstock, KW, and MSW respectively where biogas production was observed to begin within the first 8 h in cooked bananas, cooked Irish potatoes, cabbage, spinach, Irish potato peels, and banana peels; which is in accordance with [35] and it is because of the existence of more degradable compounds in those feedstock. On other hand, no gas was observed in cooked rice waste, cooked beans waste, ugali, and kale for the first 8 h due to the presence of hard degradable compounds in the feedstock [35]. The experiment was conducted only for 10 days and then production ceased except for cooked beans and rice whereby production in cooked beans ceased after 11 days but cooked rice continued until 28 days when production stopped. During anaerobic digestion of cooked rice waste and cooked beans waste, the hydrolysis and acidogenesis process occur which leads to the accumulation of intermediary by-products [35] as the growth rate of acidogenic is higher in comparison to methanogens. The acid-consuming microbes are extra sensitive, reticent by an assemblage of acid, and therefore decrease pH than the acid-forming microbes. This leads into the drop in biogas yield before the acid-consuming microbe's activity is recovered. This was observed in cooked rice waste whereby production within the first 8 days was very low and increase with the increase in time. The highest biogas yield was observed in cooked rice (2821 ± 31.03 mL) followed by cooked beans (983 ± 10.81 mL) and the lowest was in ugali (15 ± 0.17 mL). Low CN ratio and high or low biodegradability of substrates are the major constraints of anaerobic AD which leads to process hindrance due to the accumulation of VFA [36].

#### 3.3.2 Effect of total solid content on biogas production

Fig. 4(a and b) indicates the outcomes of total solid on biogas generation of different KW and MSW, the study indicates that the highest biogas production was attained in cooked rice waste having TS of 31.00% which generate 2.821 L/(g VS) for 28 days. Maamri et al. [37] study the results of total solid concentration on the amount and standard of biogas generated where the results indicated that biogas yield rate and biogas yield potential rise with the increase in total solid concentration. From the above literature [37], the total solid concentration was 12.02, 17.58, 23.28, 26.75, 35.2 g/L were 0.186, 0.189, 0.93, 0.213, and 0.231 L/(g VS), respectively. Sathish et al. [38] reported that the amount of biogas yield varies according to the TS used as 10%, 20%, and 30% was 1.130, 1.250, and 1.030 L/(g VS), respectively. Jabeen et al. [39] studied the outcomes of total solid and temperature and reported that biogas yield does not depend on total solid concentration this is in line with the current study. Therefore, based on these findings, biogas yield depends on the total solids as well as other factors includes BI, NO_3_, EC, among others.

#### 3.3.3 Effect of total volatile solids on biogas yield

Fig. 5(a and b) shows biogas yield against total volatile solids of KW and SMW substrates. Volatile solids are among the factors that contributed to the amount of biogas production, although other factors play a role as well. It depends on many factors not only VS, for instance, a study whereby reduction of VS showed the highest biogas yield [40]. Regarding the current study, cabbage is the substrate that has high TVS 96.36% and yet did not produce the highest amount of biogas as compared to cooked rice and beans as its CN ratio of 13.9 is lower than the minimum recommended value for the methanogenic bacterial. The pH for cabbage was 6.2 which leads to easy accumulation of VFA [28,41]. Hydrolysis and alcoholic formation of vegetable and fruit waste occur at a higher speed as compared to other organic food waste substrates [42] which leads to low production of biogas in cabbage. Food wastes and vegetable wastes as mono-digestion substrates had the smallest duration of producing biogas except for CR with a total solid of 31%, which produced the highest yield for 28 days. Food waste substrates are highly useful for AD due to their high biodegradability and methane yield as well as the influence of pretreatment [43] regardless of other factors like CN ratio, pH, nitrates, conductivity, and alkalinity which automatically contribute to high methane yield in cooked rice. Therefore, to enhance biogas yield from vegetable waste co-digestion with the substrate with a higher CN ratio and pH is required. Biogas composition obtained from mono-digestion of cooked rice waste was found to be 62.8% methane, 36.30% carbon dioxide, 0.1% oxygen, ammonia 75 ppm, and hydrogen sulfide 681 ppm.

### 3.4 Physical-chemical analysis

A high level of nitrate in the feedstock leads to the formation of acidic compounds [44] that lower the pH value and therefore, methanogenic bacteria cannot sustain resulting in low yield. The increase in CO_2_ concentration and decrease in methane yield resulting from the high concentration of NO_3_–N can be attributed to the inhibition effects due to the intermediate compounds due to the denitrification process and also by increasing ox-red potential with the increase in nitrate concentration [45]. Therefore, an increase in nitrate concentration resulted in the inhibition of the yield. Total dissolved solids (TDS) of the substrates showed that cooked rice waste had the highest TDS **(**Table 2**),** which may probably contribute to the highest biogas generation as in contrast to the rest of the substrates used in this study.

## 4 Conclusions

Feedstock characteristics has a major role in biogas yield. The volatile solid of cooked rice was 83%, which produces the highest amount of biogas 2821 ± 31.03 mL as compared to cabbage with the volatile solid at 96.36 ± 1.73%, which produces 800 ± 10.81 mL of biogas. This is due to the low CN ratio of cabbage 13.9:1 with a pH of 6.2, which is lower than the minimum recommended value of 20:1 and 6.5–7.4 respectively. The carbon-nitrogen ratio of cooked rice waste was noticed to be 30.9 whereas its pH was 7.2, and therefore more yield. The physical-chemical analysis of cooked rice waste, ugali, and cabbage showed that there is a direct relationship between CN ratio, biodegradability index, electrical conductivity, nitrates, and total dissolved solids with biogas yield. This study recommends that cooked rice waste can be utilized alone as a feedstock for biogas generation while other substrates studied require co-digestion to achieve the required CN ratios that will accelerate degradation and hence high methane yield. Among these substrates, biogas production from cooked rice lasted longer and this could be an important factor in commercial large-scale production of biogas.

## Author contribution statement

Register Mrosso: Performed the experiments; Analyzed and interpreted the data; Wrote the paper. Joseph Kiplagat: Contributed reagents, materials, analysis tools or data; Wrote the paper. Achisa Mecha: Conceived and designed the experiments; Wrote the paper.

## Data availability statement

Data will be made available on request.

## Additional information

No additional information is available for this paper.

## Declaration of competing interest

The authors declare that they have no known competing financial interests or personal relationships that could have appeared to influence the work reported in this paper.
